# Seven steps to enhance Open Science practices in animal science

**DOI:** 10.1093/pnasnexus/pgac106

**Published:** 2022-07-11

**Authors:** Rafael Muñoz-Tamayo, Birte L Nielsen, Mohammed Gagaoua, Florence Gondret, E Tobias Krause, Diego P Morgavi, I Anna S Olsson, Matti Pastell, Masoomeh Taghipoor, Luis Tedeschi, Isabelle Veissier, Christian Nawroth

**Affiliations:** INRAE, AgroParisTech, Université Paris-Saclay, UMR Modélisation Systémique Appliquée aux Ruminants, 75005 Paris, France; Universities Federation for Animal Welfare (UFAW), The Old School, Brewhouse Hill, Wheathampstead, Hertfordshire AL4 8AN, UK; Teagasc Food Research Centre, Ashtown, D15 KN3K Dublin, Ireland; PEGASE, INRAE, Institut Agro, 35590 Saint-Gilles, France; Institute of Animal Welfare and Animal Husbandry, Friedrich-Loeffler-Institut, Dörnbergstr. 25/27, 29223 Celle, Germany; Université Clermont Auvergne, INRAE, VetAgro Sup, UMR Herbivores, F-63122 Saint-Genes-Champanelle, France; i3S—Instituto de Investigação e Inovação em Saúde, Universidade do Porto, Rua Alfredo Allen 208, 4200-180 Porto, Portugal; Natural Resources Institute Finland (Luke), Production Systems, Latokartanonkaari 9, FI-00790 Helsinki, Finland; INRAE, AgroParisTech, Université Paris-Saclay, UMR Modélisation Systémique Appliquée aux Ruminants, 75005 Paris, France; Department of Animal Science, Texas A&M University, College Station, TX 77843-2471, USA; Université Clermont Auvergne, INRAE, VetAgro Sup, UMR Herbivores, F-63122 Saint-Genes-Champanelle, France; Institute of Behavioural Physiology, Research Institute for Farm Animal Biology (FBN), Wilhelm-Stahl-Allee 2, 18196 Dummerstorf, Germany

**Keywords:** animal research, open access, open data, open peer-review, preregistration

## Abstract

The Open Science movement aims at ensuring accessibility, reproducibility, and transparency of research. The adoption of Open Science practices in animal science, however, is still at an early stage. To move ahead as a field, we here provide seven practical steps to embrace Open Science in animal science. We hope that this paper contributes to the shift in research practices of animal scientists towards open, reproducible, and transparent science, enabling the field to gain additional public trust and deal with future challenges to guarantee reliable research. Although the paper targets primarily animal science researchers, the steps discussed here are also applicable to other research domains.

## Introduction

The knowledge produced by publicly funded research is a public good, and, as such, both the outcome of research and the evidence that supports the scientific claims (e.g. protocols, data, models, and program code) should be transparent and publicly accessible. Open Science (OS) is an umbrella of practices referring to the process of making scientific knowledge accessible, reproducible, and transparent to everyone (1). Transparency and accessibility help improve the quality and production of scientific knowledge (2). In addition to the general societal and academic benefits of OS (3), Nawroth and Krause (4) argued that OS practices could also strengthen adherence to the 3Rs (Replacement, Reduction, and Refinement) principle for ethical research on animals through the possibility of reusing protocols and data, and *via* fast dissemination of protocols and findings. However, incorporating OS practices is still relatively limited in the animal science domain. By animal science, we refer primarily to research on domestic animals, including nutritional, behavioural, and physiological aspects. Breaking the barriers to engaging with OS may require learning new skills and adopting new habits. One of the major obstacles in OS engagement is the lack of institutionalized incentives and training opportunities on OS practices. Inspired by guiding papers in the domains of psychological science (2, 5) and ecology (6), we here provide seven practical steps to encourage the adoption of OS in the animal science field. These steps do not reflect a chronological order. They are sets of actions that can be implemented together or individually. In addition, we propose answers to some common questions related to the adoption of OS practices (Table 1) and suggest engagement actions to enhance OS in the field of animal science (Table 2).

**Step 1: share your code and data**

Some research communities, such as those working in genomics and proteomics, have a long history of data sharing *via* specific repositories. However, the animal science field is still subjected to barriers to data sharing (see Table 1). If we were (more) willing and able to provide Open Access (OA) to our data, codes, and models, these resources could more easily be part of meta-analyses (7) and help ensuring the reproducibility of experiments. The “FAIR Guiding Principles for scientific data management and stewardship” (8) describe general guidelines to improve the findability, accessibility, interoperability, and reuse (FAIR) of digital assets. One of the barriers to sharing data and code is the need to guarantee that they are stored safely and are citable (assigned a Digital Object Identifier; DOI). Several open research data repositories (e.g. Zenodo, Figshare) are available to this end. The site re3data is a directory of the main data repositories. More recently, some research institutes have launched their own solutions to facilitate data sharing and open data publications for their researchers and collaborators (e.g. the Portail Data INRAE, Open Agrar). For data connected to publications, journals may impose specific repositories. In addition to data repositories, several platforms providing data services (e.g. tools for data analysis) are available such as OpenAIRE and EOSC among others.

To follow the FAIR principles, it is important to add a description of your data set in the data repository. This set of information describing a data set is called metadata. It is important to use the same terms when referring to the same variables. Ontologies have been developed for livestock phenotypic traits (ATOL), for lab analyses data (JERM), and for bioinformatics analyses (Gene Ontology).

**Table 1.** Frequently asked questions about OS.

**Questions**	**Answers**
Why should I share my data?	Sharing data enables building aggregated datasets in the long term that promotes methodological progress, validation of findings, and reproducibility. Shared datasets can also increase the interest of others towards your work and lead to more citations and new collaborations.
The development of my model was a very time-consuming task, why should I share it?	Once your paper is published, submitting the code in an adapted repository ensures that your results are reusable. Your code can also be cited. Sharing your code will increase visibility and citation of your work. Others may build upon your work and improve the method.
Do preprints provide a record of the originality of my research? If I publish a preprint, can my research be scooped?	Preprints help to establish priority of discovery (9). Preprints prevent your research from getting scooped since they represent date-stamped priority claims. However, be aware that your preprint might attract the attention of the media, which can refer to your manuscript as if it was a peer-reviewed article. Also note that a preprint is a public disclosure, and that intellectual protection depends on the license you choose when depositing the preprint (see information on licenses).
Will my preprint negatively affect the citations of my subsequent journal article?	Preprints increase the visibility of your work. Some studies show positive correlations between preprint posting (and downloads) and citation metrics of the subsequent journal paper (9). Note that Google Scholar allows you to merge preprints and published articles.
I have preregistered my work. Is it still possible to change my test protocol?	Yes, it is possible to alter your test protocol. However, these changes must have very pragmatic reasons and must be transparently outlined in the preregistration/final article. If you have submitted a Registered Report, it is best to contact the editor of your submission and get approval from the journal side.
Where can I learn about open source software development, data sharing, and other OS features?	Software Carpentry and Data Carpentry are good places to start for beginners. Getting involved in existing open source projects is also a good way to learn. The FOSTER OS project is a great resource of courses for OS training. Check if your institution offers training in adopting OS practices.

**Table 2.** How can you engage in the OS debate and break down barriers?

• Engage with your colleagues: share your experiences of OS, teach, and promote OS practices. Use this or similar articles (10) for your next journal club. Promote OS workshops in your network.
• Adopt OS practises in your team: discuss how the revenue scheme can be changed to reward OS and open-project collaborative research. Forward the questions and solutions to your group leader or head of institute.
• Engage with institutions and funders: promote the recognition of OS practices in the evaluation of job and project applications, and in our scientific societies. Discuss the positive and negative aspects of different metrics (e.g. impact factors) in science evaluation within your network (e.g. 11). Invite your institutions to engage in OS actions (12) and adopt quality research assessment indicators as proposed by the San Francisco Declaration on Research Assessment (DORA), and the Leiden Manifesto (13).
• Engage with journals: promote data and code sharing, question preprint policies, and suggest ways to improve reproducibility in particular when you are an editor. Suggest sharing of code and data in your peer-reviews even if it is not required by the journal.
• Promote bibliodiversity: explore innovative publication models that go beyond traditional journal formats. This includes data papers and submitting to alternative peer-review schemes such as Peer Community in Animal Science (see, e.g. the Jussieu call for OS and biodiversity).

After depositing your data into a repository, you can disseminate your data e.g. *via* a data paper, which is an article that describes research data in a structured and readable form. Some journals accepting data papers are CABI Agriculture and Bioscience, Animal Open Space, Scientific Data, and Data in Brief.

For source code, it is important to make your code easy for others to use. Storing your code in a public repository using an open-source license makes it freely accessible, (re)usable, and improvable by anyone. You will need to provide instructions for running your code with a reproducible example and to list required software with version details included. You can provide your method as an installable library (e.g. R or Python package), including appropriate documentation (14). To make your code and data analysis reproducible, it is recommended to follow standards on programming best practices and workflow automation as discussed by Heil et al. (15) for machine learning analyses.

Software packaged as a library can also be installed directly from a repository in many programming languages. The Software Carpentry and Data Carpentry nonprofit communities provide workshops and online lessons for adopting OS practices. Repositories such as GitHub and Sourceforge provide useful services for sharing scientific software.

Several funding agencies now encourage researchers to consider data sharing (which, when, with whom) from the start of research projects. A Data Management Plan (DMP) is now mandatory in projects funded by the European Commission, describing how data will be collected, processed, shared within the collaborators of the project, made openly accessible, and preserved. DMP templates and tools (e.g. OPIDoR, ARGOS) can be found on websites of funding agencies.

**Step 2: preprint your findings**

Peer-review ensures scientific quality. However, reviewing and the full editorial process from first submission of an article to its publication often takes several months, delaying the time to access and sharing research output (16). Accelerating the dissemination of scientific findings is particularly important for Early Career Researchers, who rely heavily on timely publication of their work to advance in their careers (17). Preprints offer a solution for this: They are published manuscripts that have not (yet) gone through formal peer-review. A DOI assignment provides an identifier with time stamp on the preprinted publication and thus can be useful to establish priority (please note that some preprint servers/repositories such as arXiv and HAL do not provide a DOI but a permanent URL). The DOI and permanent URL allow the preprint to be cited and to be findable, e.g. in Google Scholar. Preprints also provide a way of sharing scholarly content that would otherwise be lost (16). There is, however, a major drawback of the preprint model: the lack of peer-reviewing means that there is no external assessment of scientific quality of the posted manuscripts. Open peer-review (OPR) platforms partly circumvent this drawback (see Step 7). Indeed, preprints allow informal feedback from peers prior to formal peer-review and may thus help to improve manuscripts before their submission to a scientific journal where they will undergo a peer-review process.

Most journals in animal science allow preprinting (see Sherpa Romeo to check the journal policy). Preprints can be deposited in three types of platforms: (1) preprint servers like bioRxiv, arXiv, OSF; (2) institutional repositories of which many universities and institutes have their own; and (3) multidisciplinary archives like Zenodo and HAL. There are plenty of established preprint servers for life sciences, but only very few are specifically for animal science (e.g. agriRxiv). OpenDOAR is a directory of OA repositories. Once a server is chosen, the actual preprinting is relatively simple: upload your final document and add meta-data—that is it. It is best practice to upload the most recent version to the preprint server (the file will be updated, but previous versions are still accessible.) However, it should be noted that some platforms, such as agriRxiv, do not accept versioning at the moment. Once published in a journal, you should include the full reference and link to your journal article in the preprint version—both steps will facilitate access to the most recent content and ensure your work is cited properly. Services such as Google Scholar allow you to merge the preprint and the corresponding peer-reviewed journal papers, so they will not be duplicated in your publication list and will not bias the calculation of some metrics such as the h-index.

A common concern about the preprint model is the risk of proliferation of low-quality manuscripts due to lack of peer-reviewing. However, authors may be cautious to release work of low quality as this would harm their scientific reputation. Indeed, there is no indication of low-quality preprint issues in the physics, mathematics, and computer science communities as a result of the widespread placement of preprints in the arXiv server (18). The same situation seems to apply for preprints in the field of biology (see ASAPbio webpage).

**Step 3: publish your articles under OA licenses**

Making scientific publications freely available is an essential part of OS. The OA publication in scientific journals occurs mainly *via* two routes: the gold route and the green route (see Step 4). The gold route refers to the publication in journals where all the articles are published under an OA license. There are several OA journal business models including an Article Processing Charge (APC) model in which the author pays a publication fee to make their work OA. Although the APC model allows breaking the barrier imposed by journal paywalls, the high costs of APCs (up to €9500) impose economic barriers that discriminate against researchers in low- and middle-income countries.

Many so-called predatory journals use the OA system to lure researchers offering special rates or fast processing times for their manuscripts (19). Be aware, you can check dedicated websites that help you identify predatory journals (see, e.g. thinkchecksubmit and Compass to Publish).

A derivative of the gold OA route is the publication in hybrid journals, which are traditional subscription journals (with paywalls) that offer the OA option *via* APC. The business model of the hybrid journal is controversial since both authors and readers are charged (APC and paywalled subscription fees). An alternative solution to the obstacles of APCs is given by diamond (sometimes labelled platinum) OA journals, often managed by noncommercial and nonprofit societies and organizations. In these journals, publications are made OA at no cost for authors and readers, with the cost being generally covered by a financial and in-kind donation from research institutions and universities. Diamond OA journals operate often at local and national levels. The Directory of Open Access Journals (DOAJ) indexes most of the journals using OA models.

It is important to highlight that the publication landscape is multidimensional (20). For example, academic publishing can be analyzed *via* three dimensions in regard to (i) the economic model (for-profit, non-profit-commercial, non-commercial), (ii) the openness model (closed access, open access), and (iii) the ownership models (society, non-society). Whereas this paper focuses on openness, the economic and ownership models are also relevant when choosing a journal. When considering the ownership dimension as a criterion for the choice of journal, many society journals can have benefits over nonsociety journals, because part of the publication fees from these society journals is often used to support actions for the scientific community (e.g. training and grants for young scientists). Many society journals, thus, contribute to retain part of the publication fees in academia. Authors should nevertheless check the policy of specific societies individually. At the opposite side, purely commercial journals remove publication fees from the research domain. Society journals have a major role to play towards more open and ethical publishing models, for example, by gaining independency from for-profit commercial publishers. This type of action will result in lowering APCs while allowing to keep research resources within academia (20).

In 2018, the Plan S initiative for OA publishing was launched. This initiative is supported by an international consortium of organisations that fund and carry out research. Plan S requires that, from 2021, scientific publications resulting from research funded by public grants must be published in compliant OA journals or platforms.

**Step 4: upload your non-OA papers into a repository**

Often scholars and institutions do not have the funding to cover APCs to publish under a gold OA license. But even when published behind a paywall, these articles can be made OA *via* the green OA route by posting a postprint, i.e. the final version of a manuscript following peer-review but without the journal layout, to a research repository like those mentioned in Step 2. Many institutions already have OA policies that require the uploading of postprints into an institutional repository. Authors should be aware that publishers have different embargo periods for publishing these postprints (check Sherpa Romeo for the journal of your choice). Keep in mind that some countries impose constraints on these embargo periods. For example, in France, researchers are authorized to engage in green OA archiving of publications from work that has received public funds no matter the publisher's policy (embargos in France are thus set at 6 months for basic sciences and 12 months for human and social sciences). You should check the policies of your institution and country to know when and in which repository you should deposit postprints.

Publishing *via* the green OA route is not only an option to save resources when submitting a new manuscript by saving APCs, it can also be of relevance for previously published, but pay-walled, work. Years ago, authors might not have considered the additional value of making research accessible to everyone (or may not even have had this option) and do now, in hindsight, regret their choice of not publishing OA. With the green OA route, authors can also make all their previous research accessible—better late than never! Depositing postprints in repositories is also a way to preserve the scientific literature since journals can disappear from the internet. Repositories, however, can also disappear. It is therefore important to consider features like ownership, preservation, and indexing when choosing a preprint repository. Similar to preprinting, when uploading your postprint you should provide a reference and link to the full paper on the title page of your manuscript—this will make the published paper easier to find and cite.

**Step 5: preregister your study**

To ensure appropriate methods are used when collecting and analyzing data, test protocols and analysis plans should be preregistered (21). This consists of authors depositing their hypotheses and study designs on preregistration servers (e.g. OSF) before data collection begins. There are also specific registries for animal experiments, such as the Animal Study Registry and Preclinicaltrials.eu. Preregistration can be useful to prevent questionable research practices (QRPs) such as p-hacking (i.e. changing statistical methods until a *P*-value below 0.05 appears) and HARKing (Hypothesising After Results are Known) (22). Both of these are highly problematic as they can mislead researchers that a biological relevant effect exists where there is not any. The early documentation of the test and analysis protocol has the potential to increase the credibility of results and thus public trust in science. It is important to highlight that preregistration protocols, once submitted, are not set in stone—deviations, well argued, are possible and should always be disclosed (see 23 for problems with disclosure and best practice recommendations). It should be noted that preregistrations have the option to be made public only after a certain embargo period, so that the research idea will remain protected.

**Step 6: publish a Registered Report**

Preregistrations of studies (see Step 5) can also be submitted to a journal and undergo formal peer-review—with the resulting article type being called “Registered Report” (RR) (24). In general, studies resulting from RRs show much lower effect sizes, proving that biases (such as negative results not being published) have caused inflation of effects in traditional, non-preregistered published articles (25, 26).

Academics can receive a provisional acceptance by a journal based on their submitted RR, thereby streamlining the submission and publication processes. RRs should not take more time than conventional publishing, because the time spent writing and reviewing the first half of a scientific paper is simply done before the experiment is conducted. It is therefore not more work but rather a reorganization of the different steps involved in the process. An additional advantage for the authors of RRs is the possibility to get feedback at a point in the research project when the experimental design can still be improved. This can have a significant impact on the welfare of the animals used in your research: Preregistering your test protocol, and its effect on the occurrence of QRPs, can be beneficial in terms of a better alignment of your research with the 3Rs principle. Feedback on RRs can flag outdated or suboptimal test designs/paradigms to answer specific research questions, effectively lowering the number of animals used in your research in the long term (4). It also ensures that negative (lack of significant) results from animal experiments become available to the public. Especially Early Career Researchers will benefit, as acceptance of a RR can be useful for job or funding applications, as it occurs early in the research process.

Several general journals as well as some journals in the animal science domain, such as Animal Behavior & Cognition, accept RRs (see this list of journals accepting RRs). PCI Registered Reports is dedicated to RRs.

**Step 7: participate in Open peer-review**

The traditional journal evaluation process (i.e. peer-review) is often a black box where peers not involved in the process have limited insight into the discussions and criteria leading to the acceptance of published articles. Open peer-review (OPR) refers to a set of features that makes the full process of evaluation of scientific articles (including preprints, see Step 2) open and transparent (27). Here, we focus on the feature of making public the review reports by peers—a practice that strengthens the scientific debate, increases accountability, and reproducibility, and contributes to the development of constructive peer-review. In addition to promoting transparent and constructive debate, OPR acknowledges the work carried out by editors and reviewers (if they decide to be named). Platforms like Publons give peer-review recognition (see a comment about peer-review recognition) and allow reviewers to make their reviews public if they wish.

For journals adopting OPR, the reviewer reports and the editorial decision are made public when the paper is published. The journal implementation of OPR is often made as an optional feature for the authors and reviewers. Some multidisciplinary journals like PLoS One, eLife, and Royal Society Open Science have implemented the OPR option. To our knowledge, no disciplinary journals in animal science have yet adopted OPR within their evaluation system. The OS initiative Peer Community In (PCI) Animal Science and the Peer Community Journal, which belong to the parental project Peer Community In, provide a free process of OPR evaluation of preprints (see an example of OPR by PCI Animal Science).

OPR can also take place after the publication of a manuscript by tools developed by journal publishers (e.g. *Frontiers In*) or *via* independent platforms. These platforms can be used for the evaluation of published articles and preprints. Some examples are PREreview, F1000Research, Review Commons, Hypothes.is, and PubPeer.

We encourage you to adopt OPR in your research practices as both author and reviewer. As reviewer, you can go further and align with the Peer Reviewers' Openness Initiative (28) by providing comprehensive reviews of a manuscript only if it meets minimum OS requirements (e.g. the data should be made publicly available). Keep in mind that making the identity of reviewers open is a controversial issue. For example, Early Career Researchers may not want to go public when reviewing work by more established scientists.

## Conclusions

The OS umbrella covers several practices aiming at making scientific knowledge accessible, reproducible, and transparent. We provide here an overview of seven steps to better embrace OS in animal science research. These steps are also applicable to other research domains. Most of the actions are relatively easy to put into practice (e.g. depositing a manuscript in a preprint server takes less than 5 minutes. You can start today!). Other actions require certain expertise and effort (e.g. building a DMP). We encourage you to adopt the OS practices presented here in your research. Start progressively, choosing those steps that you feel more comfortable with as a start, keeping in mind that the time you spend on implementing OS practices will be rewarded. As we expect OS to become *the new normal* in our field, this can only be possible by our active engagement (Table 2).

## Notes

**Competing Interests:** We established as conflict of interest the involvement of authors in journal boards or Open Science initiatives mentioned in the manuscript. All authors but E.T.K. are members of the Open Science initiative Peer Community In Animal Science. I.A.S.O. is an academic editor for *PLoS One*. B.L.N. is an academic editor for *Frontiers in Veterinary Science*. C.N. is an academic editor for *Animal Behaviour & Cognition* and *Frontiers in Veterinary Science*. D.P.M. is an academic editor for*Frontiers in Microbiology*. R.M.T. is a member of the scientific board of *Animal*.
